# Listening in Reverse: A Case for Retrograde Signaling During Auditory Inner Hair Cell Ribbon Synapse Maturation

**DOI:** 10.3390/cells15191779

**Published:** 2026-09-29

**Authors:** Roos A. Voorn, Louisa S. Kuenkel, Jan F. Ahrend, Christian Vogl

**Affiliations:** Auditory Neuroscience Group, Institute of Physiology, Medical University Innsbruck, 6020 Innsbruck, Austria

**Keywords:** auditory pathway, transsynaptic signaling, cochlear development, endocannabinoids, nitric oxide, cell adhesion molecules

## Abstract

Cochlear inner hair cells (IHCs) conduct rapid, sustained, and temporally precise transmission of acoustic information onto spiral ganglion neurons (SGNs). For this purpose, IHCs utilize highly specialized sensory synapses. During development, these so-called ‘ribbon’ synapses and their postsynaptic counterparts undergo extensive structural and functional refinement—a process driven by pre-sensory spontaneous activity. The underlying molecular mechanisms remain incompletely understood to date. Interestingly, little is known about the involvement of retrograde feedback provided by postsynaptic SGN terminals to transsynaptically instruct ribbon morphology and presynaptic active zone organization. This review therefore aims to shed light on this phenomenon and assess a potential role of retrograde signaling during IHC ribbon synapse maturation. We first summarize the main mechanisms established for conventional CNS synapses before discussing the current evidence regarding these pathways at IHC-SGN synapses. In particular, we focus on the gasotransmitter nitric oxide, lipid-derived endocannabinoid signaling, and cell adhesion molecules as the most promising candidate pathways. In summary, we propose that differential retrograde signaling may contribute to IHC synaptogenesis, SGN subtype diversification as well as the pruning of afferent contacts during development and might even play an extended functional role in auditory ribbon synapse regulation after hearing onset.

## 1. Introduction

**Figure 1:**
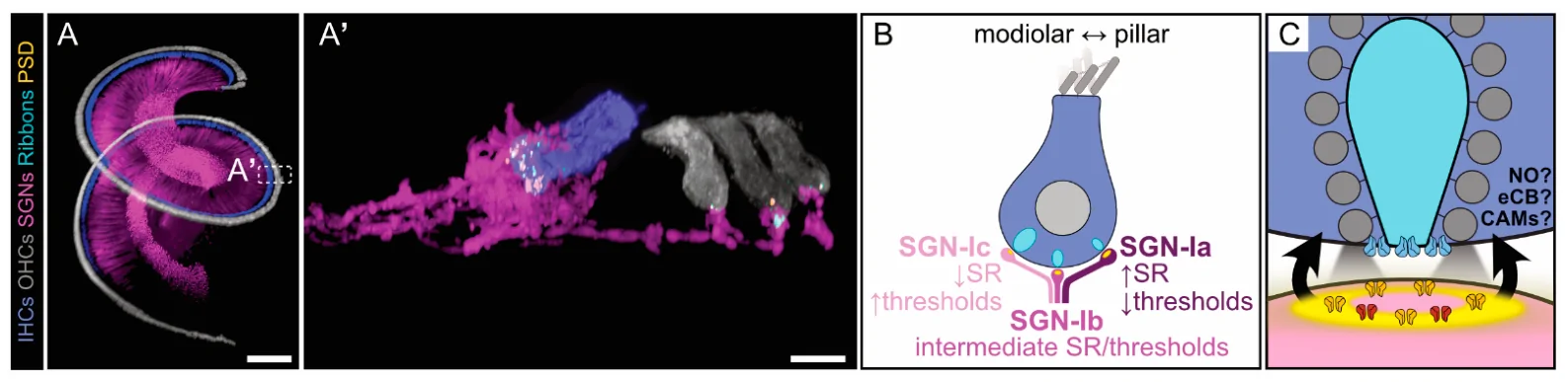
**Anatomy of the peripheral auditory pathway.** Representative lightsheet fluorescence microscopy image of the isolated peripheral auditory system, immunolabeled for inner hair cells (IHCs, blue), outer hair cells (OHCs, gray), and afferent spiral ganglion neurons (SGNs, magenta). (**A’**) Representative close-up confocal maximum projection of hair cell innervation in the organ of Corti, with additional immunofluorescent labeling of presynaptic ribbons (cyan) and postsynaptic density (PSD, yellow). (**B**) Schematic illustration of the three SGN-I subtypes (a, b, c) innervating an IHC, with focus on their differences in (i) distribution along the IHC modiolar–pillar axis, (ii) morphology, including the size of presynaptic ribbon and postsynaptic AMPAR cluster, and (iii) functional features, such as spontaneous firing rate (SR) and activation threshold. (**C**) Schematic of an IHC-SGN type I synapse: the IHC presynaptic active zone contains a synaptic ribbon (cyan) that tethers synaptic vesicles (gray), and clusters voltage-gated calcium channels (light blue), while the SGN terminal contains a halo-shaped PSD (yellow) that harbors AMPA and NMDA receptors. Black arrows indicate some of the main retrograde signaling mechanisms that may act here: nitric oxide (NO), the endocannabinoid (eCB) system and cell adhesion molecules (CAMs). Scale bars: (**A**) 200 µm, (**A’**) 10 µm.

Upon developmental assembly, mammalian excitatory synapses undergo an extensive maturational refinement process to achieve adequate performance. This developmental plasticity is of fundamental importance to structurally stabilize pre- and postsynaptic compartments and ensure utmost efficiency of excitation–secretion coupling. Moreover, while essential contacts are strengthened in an activity-dependent manner, less important or redundant ones are either weakened or even entirely eliminated during maturational pruning. Recent years have furthered our understanding of these processes at conventional neuronal synapses; however, much less is known about the developmental refinement of specialized high-throughput sensory synapses, such as the ones formed between auditory inner hair cells (IHCs) and afferent spiral ganglion neurons (SGNs) that facilitate synaptic sound encoding of acoustic stimuli in the peripheral auditory pathway (reviewed in [1,2]) (Figure 1A,A’).

Mature auditory ribbon synapses are monosynaptic connections capable of transmitting acoustic information with millisecond precision and at frequencies of hundreds of Hertz. To achieve such remarkable performance, IHC presynaptic active zones (AZs) are equipped with proteinaceous electron-dense bodies, so-called ‘synaptic ribbons’, which primarily comprise the structural cytomatrix protein ‘RIBEYE’ [3]. Ribbons provide presynaptic scaffolding, cluster presynaptic voltage-gated Ca_v_1.3 Ca^2+^ channels at the AZ membrane and tether a halo of synaptic vesicles (SVs) (Figure 1A’–C). This latter feature is thought to ensure ultrafast and sustained vesicular replenishment to the release site during periods of recurrent or persistent stimulation. On the postsynaptic side, ribbons are juxtaposed to large, often ring-shaped, ionotropic glutamate receptor (iGluR) clusters that consist of GluA2, GluA3 and GluA4-containing α-amino-3-hydroxy-5-methyl-4-isoxazolepropionic acid (AMPA) and heteromeric N-methyl-D-aspartate (NMDA) receptors (AMPAR/NMDAR). During development and beyond, these iGluRs act in concert to enable efficient information transfer from the pre- to the postsynaptic compartment.

Apart from the well-established role of AMPA- and NMDA-type iGluRs in anterograde signal transmission at IHC ribbon synapses, the existence and developmental role of local activity-dependent retrograde feedback loops remain to be explored. In this context, it was shown recently that IHC-mediated glutamate release plays a critical role in downstream SGN subtype diversification [4,5] and hence acts as a major driver of auditory pathway maturation. The three identified subtypes, SGN type Ia, Ib and Ic, have preferred locations on the IHC pillar/modiolar axis and are characterized by distinct morphological and functional properties (Figure 1B). Type Ia synapses connect to the IHC pillar face and harbor small ribbons that are opposed to large postsynaptic AMPAR clusters. Such fibers exhibit high spontaneous firing rates with low thresholds of activation. In contrast, type Ic fibers preferentially contact the modiolar side of IHCs, have larger ribbons and comparably small postsynaptic AMPAR patches. Functionally, Ic fibers display low spontaneous rates with high thresholds [6,7]. Finally, type Ib fibers exhibit a somewhat mixed molecular profile that shares features of Ia and Ic fibers. Interestingly, genetic deletion of the neuronal transcription factor *Runx1* disrupted the SGN subtype composition and led to an over-representation of type Ia fibers [8]. This populational shift also led to a breakdown of the pre- and postsynaptic ribbon as well as AMPAR patch size gradients. Moreover, conditional deletion of the neuronal transcription factor and SGN type Ic marker *Pou4f1* led to altered Ca_v_1.3 activation properties and increased voltage sensitivity at IHC presynaptic active zones [9]. Both findings are hence compatible with transsynaptic signaling mechanisms taking place at IHC-SGN synapses that retrogradely instruct synapse formation, functional properties and/or developmental structural plasticity. However, albeit a fascinating research topic, remarkably little is currently known about the existence and maturational role of retrograde feedback mechanisms at auditory ribbon synapses (Figure 1C).

In this review, we therefore aim to provide a concise overview of putative retrograde SGN to IHC signaling mechanisms that may contribute to ribbon synapse maturation in the cochlear periphery. For this purpose, we will briefly summarize the fundamental basics of the main retrograde signaling pathways at CNS synapses, before reviewing the state of knowledge regarding the presence of the respective critical molecular components at IHC afferent synapses and hence determine the likelihood of their potential contribution to auditory ribbon synapse maturation. Thereby, we identify shortcomings in the current state of knowledge and propose targets for future research. To meet this end, we screened the available cochlear literature but also revisited publicly available single-cell RNA sequencing (scRNAseq) data from various pre- and postnatal ages for SGNs (i.e., embryonic (E)15.5, postnatal (P)1, P8, P14, P30) as well as hair cells (i.e., P12) from Li and colleagues [10] to identify relevant developmental expression patterns.

For the purpose of providing a complete picture, it is worth noting that besides the herein proposed putative retrograde regulation of the afferent pathway, developing IHC ribbon synapses also undergo extensive efferent regulation from the auditory brainstem. Here, medial olivo-cochlear fibers release acetylcholine and gamma-aminobutyric acid (GABA) onto IHCs to pace their rhythmic spontaneous activity [11,12,13], whereas lateral olivo-cochlear fibers utilize a cocktail of classical neurotransmitters and various neuropeptides to additionally support SGN maturation and protect against noxious noise in adulthood [14,15,16]. However, since efferent influence on early auditory development has been reviewed in detail elsewhere, it will not be further addressed here [17,18,19].

## 2. A Brief Overview of Retrograde Signaling Pathways at Conventional Neuronal Synapses

**Figure 2:**
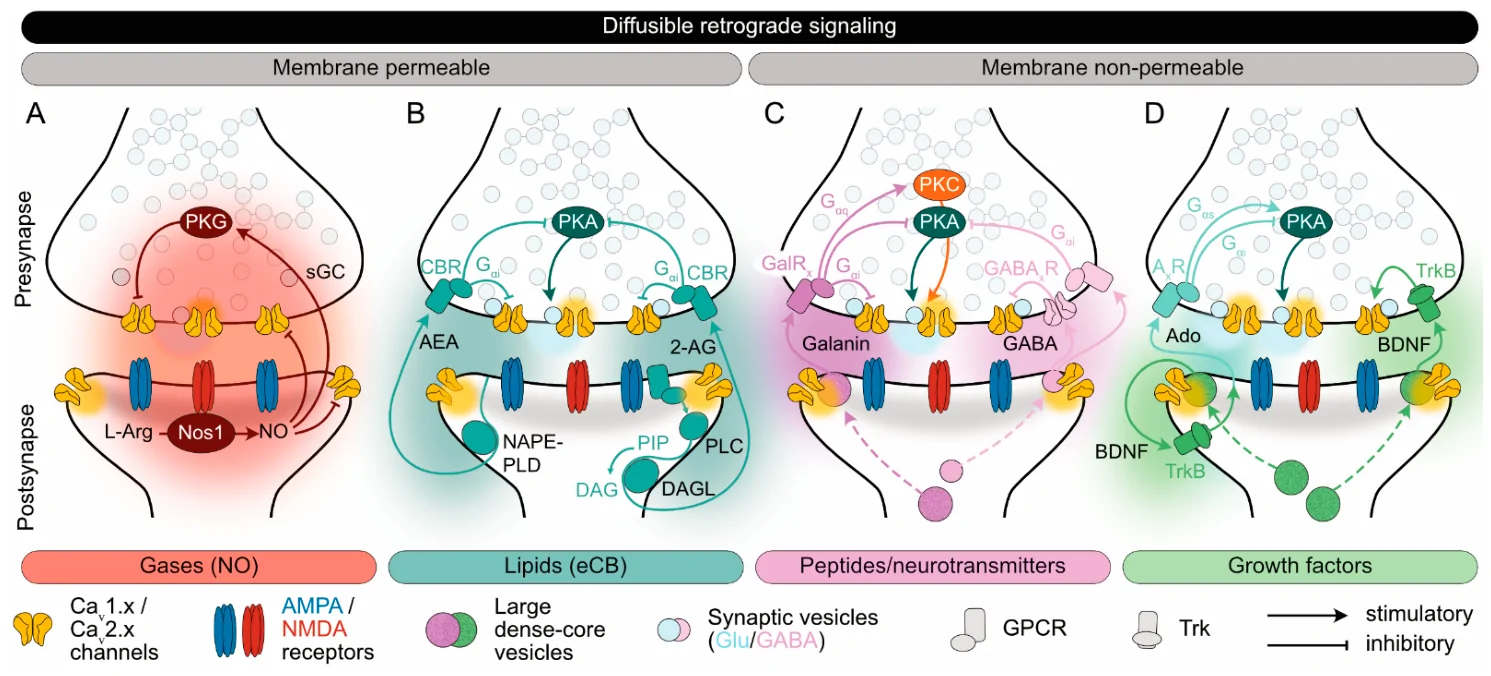
**Schematic overview of the main diffusible retrograde signaling systems at CNS synapses according to messenger class.** Only the most relevant compounds are represented for each type. Diffusible systems are initiated via postsynaptic depolarization-dependent Ca^2+^ influx, mediated via NMDARs or Ca^2+^-permeant AMPARs. Membrane-permeable messengers (**A**,**B**) are generated on-demand and diffuse freely into the synaptic cleft, while membrane-non-permeable ones (**C**,**D**) are liberated via vesicular exocytosis. (**A**) Gaseous messengers—focus on NO. Postsynaptic NMDAR-mediated Ca^2+^ influx leads to Nos1-mediated production of NO, which can decrease presynaptic neurotransmitter release indirectly by triggering sGC-mediated PKG activation or by directly modulating pre- as well as postsynaptic targets, including various ion channels and receptors. (**B**) Lipid-derived messengers, focus on eCBs AEA (left) and 2-AG (right). Postsynaptic Ca^2+^ influx and/or activation of type I mGluRs triggers the synthesis of 2-AG by PLC and DAGL, while AEA is synthesized by NAPE-PLD. eCBs can bind to presynaptic CBRs, leading to inhibition of presynaptic Ca_v_s and PKA activity. (**C**) Peptides/neurotransmitters, focus on galanin (left) and GABA (right). Postsynaptic release occurs via large dense-core vesicles (LDCVs) or synaptic vesicles, respectively. On the presynapse, galanin activates GalR_x_, leading to either attenuated release via Gα_i/o_-mediated inhibition of Ca_v_s and/or PKA or stimulatory activation of PKC, while GABA binds to ionotropic GABA_A_R or metabotropic GABA_B_R, thereby hyperpolarizing the terminal and reducing PKC activity, respectively. (**D**) Growth factors—focus on BDNF. Being released from the postsynapse via LDCVs, BDNF can either trigger postsynaptic Ado production and release (autocrine, left), which then binds to presynaptic A_x_R to initiate either stimulatory or inhibitory effects on the presynapse, or it binds to presynaptic Trk_B_ (paracrine, right) where it facilitates neurotransmitter release. Ado, adenosine; BDNF, brain-derived neurotrophic factor; CBRs, cannabinoid receptors; DAGL, diacylglycerol lipase; eCBs, endocannabinoids; LDCVs, large dense core vesicles; NAPE-PLD, N-acyl-phosphatidylethanolamine-specific phospholipase D; NO, nitric oxide; Nos1, neuronal nitric oxide synthase; PKA, protein kinase A; PKC, protein kinase C; PKG, protein kinase G; PLC, phospholipase C; sGC, soluble guanylyl cyclase; SVs, synaptic vesicles; TrkB, tropomyosin receptor kinase B; Ca_v_1.x/2.x, voltage-gated calcium channels.

**Figure 3:**
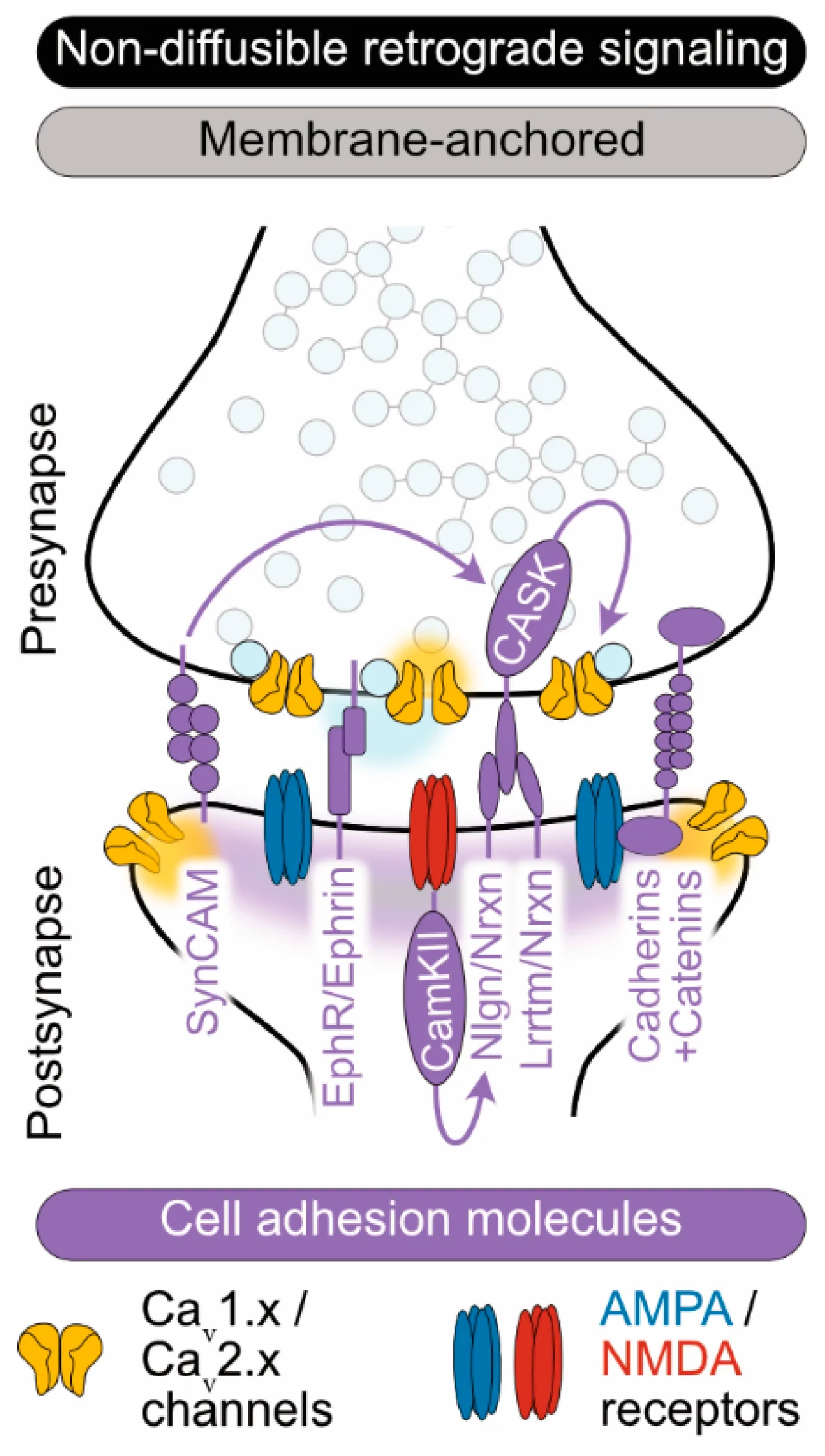
**Schematic overview of the main non-diffusible retrograde signaling systems at CNS synapse.** Cell adhesion molecules functionally connect the pre- and postsynapse—focus on SynCAMs, EphR/ephrin, Nrxn/Nlgn, Nrxn/Lrrtm and cadherins. Synaptic activity can trigger changes in postsynaptic volume, spatial organization or enzymatic state; through the transsynaptic bridge of CAMs this can affect SV release probability, AZ reorganization, protein recruitment and expression levels. Highlighted here is the well-known pathway Nrxn/Nlgn, which involves CASK as main organizer at the presynapse, and postsynaptic messenger CAMKII that, amongst numerous other functions, regulates Nlgn surface expression based on NMDAR activation.

During maturation, synaptic pruning is an essential developmental mechanism to eliminate weak or inappropriately formed contacts and thus optimize the efficiency of neuronal circuits. In this process, retrograde instructions from the postsynaptic to the presynaptic terminal can act as a powerful regulatory tool, which can be subdivided into two fundamental transsynaptic signaling pathways that either act via (i) diffusible molecules that are either locally produced on-demand or stored and released from vesicles—such as gases, lipids, (neuro)peptides, neurotransmitters and growth factors (Figure 2)—or (ii) cell adhesion complexes (Figure 3). We will now provide a brief overview of the current state of knowledge at conventional neuronal synapses. For more exhaustive descriptions of the individual pathways, we refer the interested reader to a range of comprehensive reviews on these topics [20,21,22,23,24].

### 2.1. Gasotransmitters

At many central and peripheral synapses, nitric oxide (NO), a highly reactive gaseous second messenger molecule, is locally produced on-demand by neuronal NO synthase (nNOS/Nos1) within the postsynaptic compartment. This process is highly activity-dependent [22,25] (Figure 2A). Here, the close spatial proximity between Nos1 and Ca^2+^-permeant postsynaptic NMDARs is ensured by PSD93/95-mediated scaffolding [26,27,28]. Upon production, NO freely diffuses in aqueous as well as lipidic media and hence has the capacity to react with membrane-bound as well as intracellular targets by two separate mechanisms: (i) direct posttranslational S-nitrosylation of target proteins or (ii) indirect modulation via the recruitment of soluble guanylyl cyclase (sGC) and downstream cGMP-dependent activation of protein kinase G (PKG) (reviewed in [29]). Both mechanisms have been shown to differentially regulate a plethora of pre- as well as postsynaptic molecular targets, ranging from various types of voltage-gated ion channels (e.g., Na_v_, K_v_, Ca_v_, HCN and large conductance BK potassium channels) to voltage-insensitive small conductance SK potassium channels and neurotransmitter receptors (e.g., GluA2, NMDAR). Together, NO signaling hence serves to finetune cellular excitability and synaptic function [29]. While NO is widely recognized as an important regulator of synaptic plasticity at mature synapses (e.g., [30,31,32]), it has also been shown to be of fundamental importance for excitatory as well as inhibitory synapse development in the CNS [33,34]. Due to its activity-induced production, NO effects are spatially as well as temporally highly confined and can thus be estimated to act on a single synapse level under ‘normal’ synaptic activity conditions [22]. However, strong synchronous activity of nearby terminals can also result in cooperative volume transmission that extends its modulatory reach within the tissue context [35].

Next to NO, other gaseous transmitters have also been described—including CO and H_2_S, which will however not be discussed here in detail (for review, see [36]).

### 2.2. Lipid-Derived Messengers: Endocannabinoids (eCBs)

To date, probably the most investigated and hence best-described retrograde signaling pathway is the lipid-derived endocannabinoid (eCB) system (reviewed in [24]), in which two main endogenously produced ligands regulate synaptic strength: 2-arachidonoylglycerol (2-AG) and N-arachidonoylethanolamine (anandamide; AEA) (Figure 2B). Due to their lipophilic nature, eCBs cannot be stored efficiently and hence are transiently produced by distinct synthesis pathways for immediate use. In case of 2-AG, the combination of activity-dependent postsynaptic Ca^2+^ influx and activation of G_αq/11_-coupled class I mGluRs leads to downstream activation of phospholipase C (PLC). PLC then converts the plasma membrane phospholipid PIP2 into the second messenger diacylglycerol (DAG) and inositol trisphosphate. DAG is then further metabolized by diacylglycerol lipase (DAGL) to generate 2-AG, which is liberated into the synaptic cleft to interact with the presynaptic cannabinoid receptors CB1R or CB2R. Presynaptic CBR activation then triggers a Gα_i/o_-dependent signaling cascade in the terminal that ultimately downregulates synaptic release probability via (i) direct G_βγ_-mediated inhibition of presynaptic voltage-gated Ca^2+^ channels (Ca_v_s) with simultaneous activation of G-protein-coupled inward rectifier potassium channels (GIRKs) to reduce Ca^2+^ influx and hyperpolarize the membrane potential and (ii) the downregulation of adenylyl cyclase (AC)-dependent protein kinase A (PKA) activation, which increases the open probability of presynaptic Ca_v_s via channel phosphorylation. PKA activation has additionally been shown to expand the size of the readily releasable pool of SVs at hippocampal mossy fiber boutons [37,38,39,40,41,42]. Besides 2-AG, AEA is the second major eCB that is generated upon neuronal stimulation through a separate Ca^2+^-dependent biosynthesis pathway [43]: the generation of AEA from N-acyl-phosphatidylethanolamine (NAPE) is catalyzed by phospholipase D (NAPE-PLD) in the postsynaptic terminal. AEA is then released to act on presynaptic CBRs, which then again initiate Gα_i/o_ signaling to inhibit or dysregulate the above-mentioned presynaptic components.

eCB modulation of synaptic strength has been observed at both, excitatory and inhibitory synapses and is commonly referred to as *depolarization-induced suppression of excitation*/*inhibition*, respectively [44,45,46]. However, it is worth noting that a growing body of evidence indicates that eCB signaling is not inhibitory in nature per se and can also serve as a positive modulator in synaptic potentiation at certain synapses [47]. Next to the classical signaling via CBRs, eCBs also exert modulatory effects on a variety of transient receptor potential channels (e.g., TRPV1, TRPA1, TRPM8), orphan GPCRs (e.g., GPR18, GPR55) and store-operated Ca^2+^ channels (e.g., Orai1/Stim1) (reviewed in [48]). Finally, next to their receptor-mediated signaling, recent work suggests receptor-independent eCB effects via local modification of the lipid bilayer elasticity that may mediate knock-on effects on the membrane proteins within the affected microenvironment [49].

### 2.3. Vesicular Release of (Neuro)Peptides, Conventional Neurotransmitters and Growth Factors

Anterograde information transfer at chemical synapses is mediated by the coordinated presynaptic release of neurotransmitter-filled vesicles. Similarly, some forms of activity-dependent retrograde signaling utilize exocytosis of vesicular content for signal transfer. Here, postsynaptic release of either secretory granules—i.e., large dense core vesicles (LDCVs) that contain neuropeptides or growth factors—or conventional neurotransmitter-filled SVs have been shown to regulate synaptic strength (Figure 2C,D). Analogous to anterograde SV release, these mechanisms rely on a common vesicular release machinery, which consists of depolarization-dependent Ca^2+^ influx via high-voltage-activated L- or N-type Ca_v_s and/or Ca^2+^-permeant iGluRs as well as soluble N-ethylmaleimide-sensitive factor attachment receptor (SNARE) complexes to facilitate fusion with the plasma membrane.

In the context of retrograde signal transmission, neuropeptides exert their modulatory effects predominantly via presynaptic GPCR activation (Figure 2C Left). Here, classical examples include somatodendritic release of galanin-containing DCVs from *locus coeruleus* neurons that strengthens the autoinhibitory effect of noradrenaline via auto-/paracrine modulatory signaling [50,51], or postsynaptic release of dynorphin from hippocampal granule cells, which acts on presynaptic κ-opioid receptors to inhibit Ca^2+^ influx and reduce vesicular release probability [52]. While both of these examples are inhibitory in nature, it is important to keep in mind that GPCR downstream signaling strongly depends on the exact receptor subtypes that are expressed, their tendency for heteromerization with other GPCR subtypes and the promiscuity of the respective GPCR family regarding their G protein coupling [53]. Hence, it is difficult to predict what the exact physiological implications of neuropeptide signaling might entail in a given system and therefore needs to be experimentally tested on a case-by-case basis. For example, while galanin receptors Gal_1_R and Gal_3_R generally trigger inhibitory G_αi/o_ signaling, Gal_2_R can either activate repressive G_αi/o_ or stimulatory G_αq_ cascades to inhibit or boost intracellular free Ca^2+^ levels and interfere with PKA or PKC activation, respectively [54].

Next to neuropeptides, conventional neurotransmitters can also be released from the postsynaptic compartment upon adequate stimulation (Figure 2C Right), where they may initiate excitatory or inhibitory phenomena. For example, at glutamatergic neocortical synapses, dendritic release of GABA induces retrograde synaptic depression upon burst stimulation that is mediated via metabotropic GABA_B_R [55]. Conversely, in the medial habenula-interpeduncular nucleus, activity-dependent activation of postsynaptic Ca^2+^-permeant AMPAR initiates the retrograde release of GABA, which—again via GABA_B_R activation—leads to a long-lasting potentiation of presynaptic glutamate release [56]. Similarly, somatodendritic release of glutamate has been shown to activate presynaptic ionotropic and/or metabotropic GluRs in various systems, which ultimately can trigger complex positive as well as negative synaptic feedback loops. In some cases, this may additionally involve autocrine mGluR1 activation and downstream eCB signaling [57,58,59,60].

Finally, it has been shown that various growth factors, such as brain-derived neurotrophic factor (BDNF) or neurotrophin-3 (NT3), can transsynaptically influence neurotransmission (Figure 2D). In a classical retrograde signaling scenario, activity-dependent postsynaptic fusion of BDNF granules has been shown to activate presynaptic tropomyosin receptor kinase B (TrkB) and thereby enhance neurotransmitter release at a range of glutamatergic [61,62,63,64] as well as GABAergic synapses [65,66]. Yet, a more complex BDNF/TrkB signaling pathway can be present in parallel: depending on the synaptic location of TrkB, BDNF can also act in an autocrine manner to activate TrkB directly at the postsynaptic density [67]. This process promotes the activity-dependent catabolism of ATP into adenosine within the terminal, which upon its release into the synaptic cleft activates presynaptic adenosine-binding GPCRs (A_x_R). Depending on their respective subtype, this triggers an either stimulatory or inhibitory G protein signaling cascade in the presynapse to facilitate or attenuate synaptic release in a PKA-dependent manner [67]. However, also A_x_R-dependent signaling is highly pleiotropic [53], as already mentioned above for other GPCRs, and should therefore always be experimentally determined in the respective tissue context. In this latter context, it is necessary to emphasize that acute BDNF release can, even at the same synapse, occur from both sides of the synaptic cleft and, hence, does not always act as a retrograde messenger per se [68].

### 2.4. Cell Adhesion Molecules (CAMs)

Within the synaptic cleft, various cell adhesion molecules (CAMs) form a physical, transsynaptic bridge to functionally connect the pre- and postsynaptic compartments (Figure 3). During development, different CAMs fulfill roles in axon guidance, synapse establishment and specification [69]. CAMs contain intracellular interaction domains as well as crucial extracellular domains, which can transsynaptically connect to one specific (or several different) CAMs, or presynaptic GPCRs [69,70]. In addition to the structural connection and anterograde modulatory functions in synaptic transmission, various CAMs have been implicated in retrograde signaling at excitatory synapses, such as neurexins (Nrxns), neuroligins (Nlgns), cadherins, synaptic cell adhesion molecules (SynCAMs), extracellular leucine-rich repeat fibronectin type III domain-containing proteins (Elfins), and EphR/ephrins [69,70,71,72,73]. In such cases, the retrograde signal can be evoked in a multitude of different ways, including: (i) presynaptic recruitment and differential intracellular protein interaction at the presynaptic AZ [74,75,76,77,78], (ii) the initiation or dissociation of transsynaptic nanocolumns with protein condensates at the pre- and postsynapse [79,80,81], (iii) direct interaction of postsynaptic CAMs with presynaptic GPCRs or protein phosphatases [82,83], or (iv) indirect interplay with other retrograde signaling pathways (such as eCB or mGluR-mediated signaling) to trigger, for example, alternative splicing or changes in presynaptic release probability [84,85,86]. In this manner, activity-induced changes in the postsynapse, such as volume increase upon long-term potentiation (LTP), are connected to a signaling cascade that ultimately regulates presynaptic scaling and strength. For example, at conventional glutamatergic synapses, Nrxn has been shown to regulate presynaptic SV release probability and Ca^2+^ channel clustering via Nlgn1, CASK and its various presynaptic interaction partners [87,88,89,90,91]. This is a process driven by activity-induced postsynaptic augmentation of PSD95 and the associated iGluR complement [92,93]. In line with these findings, NMDAR-mediated activation of Ca^2+^/calmodulin-dependent protein kinase II (CAMKII) has been shown to induce the formation of postsynaptic condensates that re-organize receptors, scaffolds and CAMs and, through the transsynaptic connection, trigger alterations also in the nanodomain organization of the presynapse that is thought to align and optimize synaptic transmission [79,80,81]. Apart from this direct role in influencing presynaptic release, the β-Nrxn isoform has also been shown to suppress the inhibitory feedback of the eCB system in hippocampal neurons by interfering with the postsynaptic 2-AG synthesis pathway [84].

## 3. Putative Retrograde Signaling Pathways at Auditory Ribbon Synapses

In the context of auditory ribbon synapse maturation, one attractive aspect of activity-dependent retrograde signaling is the flexibility it offers for site-specific regulation of individual synaptic contacts (Figure 1). Such differential regulation could contribute to setting up the observed morphological as well as functional heterogeneity of IHC active zones [7,94,95] as well as activity-dependent SGN subtype diversification [4,5] and thus finetune the establishment of the extraordinarily wide dynamic range of sound intensity coding that is characteristic for mammalian hearing. In the following, we want to focus on the (in our opinion) most promising candidate retrograde signaling pathways in this context, namely NO-, eCB- and CAM-mediated signaling. While there is abundant evidence for the developmental importance of neurotrophic factors (such as BDNF and NT3) in SGN survival and neurite outgrowth (reviewed in [96]), the picture is much less clear for their potential role in activity-dependent retrograde neurotransmission and synaptic plasticity at the hair cell synapse. The same holds true for conventional neurotransmitters. At this point, we certainly cannot fully exclude a potential contribution of these pathways; however, we deem it rather unlikely: in published EM micrographs of IHC ribbon synapses, neither small and clear SVs nor LDCVs are commonly observed in relevant quantities within the postsynaptic terminal to manifest a major signaling pathway. Therefore, we believe that this topic should better be addressed by dedicated ultrastructure labs upon careful inspection of their available EM datasets.

### 3.1. Nitric Oxide Signaling in the Cochlea

**Figure 4:**
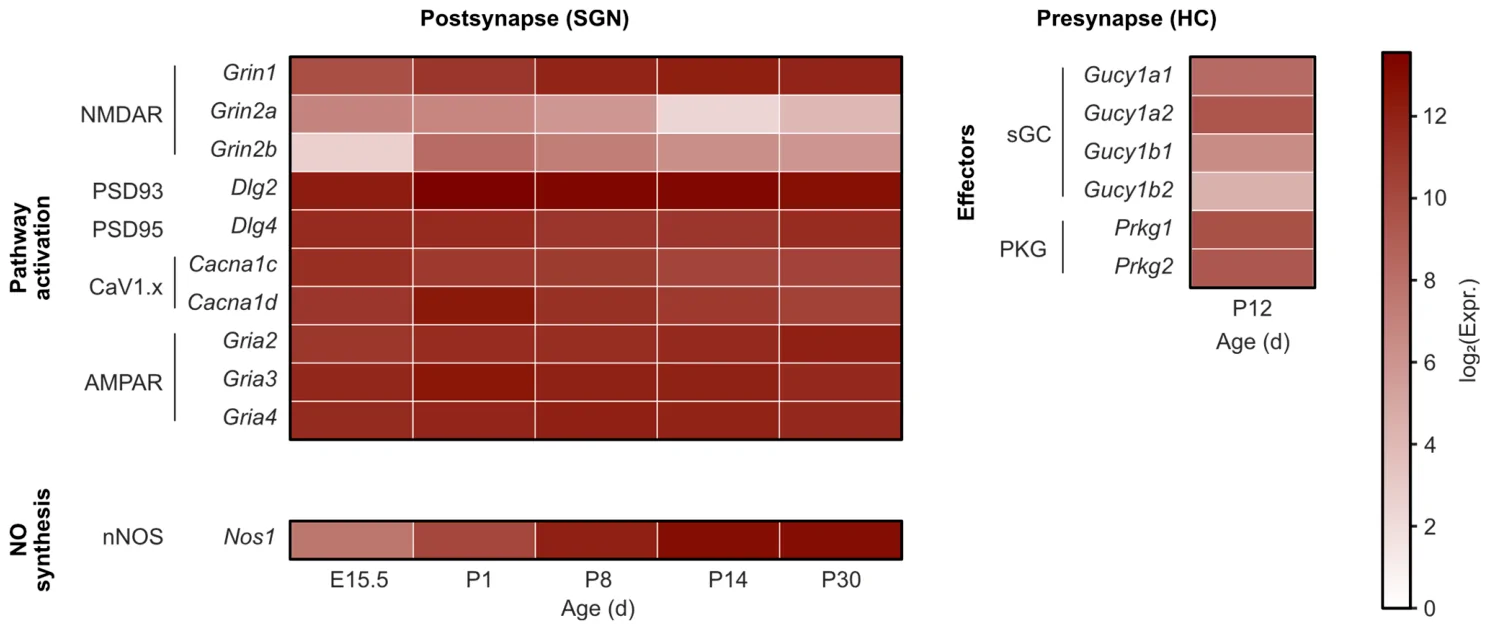
**Differential expression of selected genes associated with retrograde nitric oxide (NO) signaling in postsynaptic spiral ganglion neurons (SGNs) and presynaptic hair cells (HCs).** Data highlight cell-type-specific enrichment patterns to estimate a potential contribution of NO-mediated retrograde communication to HC–SGN synapse maturation. Genes encode components related to pathway activation, NO synthesis and downstream effectors. Color intensity represents transcript abundance. Data was acquired using bulk RNA sequencing of manually picked SGNs at different developmental time points (E15.5, P1, P8, P14, P30) and FACS-sorted hair cells at P12 (both cochlear and vestibular) [10].

NO signaling has previously been shown to acutely regulate activity-dependent synaptic plasticity throughout the ascending auditory pathway [32,97,98]. In the cochlea, Nos1 as well as functional NMDARs are widely expressed in type I SGNs [99,100,101,102,103,104,105,106]. Interestingly, *Nos1* mRNA levels seem to increase with age (between E15.5 and P30) in developing mouse SGNs; whereas genes for postsynaptic PSD93 and -95 scaffolds, Ca_v_1.x and AMPARs remained largely stable over time in the same experiment (Figure 4). This observation hence supports a maturation-related expression pattern for *Nos1*. Regarding NMDAR function, local perfusion of CPP (a commonly employed NMDAR antagonist) inhibited evoked postsynaptic Ca^2+^ transients in a subset of ~60% of young postnatal SGN terminals [107]. These data thus indicate physiological diversity and a variable degree of NMDAR functional expression that is a fundamental requirement for downstream Nos1 activation.

So how could NO signaling be of physiological importance during auditory ribbon synapse maturation? Developing IHCs spontaneously release glutamate onto afferent SGN terminals [108,109,110,111]. This process is essential for the establishment of tonotopy prior to hearing onset [112]. Hence, differential retrograde NO signaling from specific postsynaptic terminals may transsynaptically regulate the release capacity of a given ribbon synapse and thereby site-selectively instruct the pre- and postsynaptic morphology and, hence, exocytic output. Such a mechanism would be of particular importance for a compact and isopotential cell type, such as cochlear IHCs, and allow for localization-dependent and synapse-specific adjustments to synaptic transmission. In further support of this hypothesis, previous work on bullfrog hair cells, mammalian vestibular hair cells and adrenal chromaffin cells indicates NO-dependent inhibition of L-type Ca_v_1.3 via NO/cGMP/PKG and/or direct channel S-nitrosylation [113,114,115]. These mechanisms would hence translate into attenuated excitation/secretion coupling at the affected IHC active zones and could trigger compensatory homeostatic scaling. Besides Ca_v_1.3 itself, the functionally coupled SK channels have also been shown to be regulated by NO [116] and would thus present an additional important substrate for nitrergic modulation at developing IHC presynaptic AZs. During a transient postnatal period in mice, SKs assist in determining the shape and frequency of Ca^2+^-dependent action potentials in immature IHCs, and thereby directly influence their intrinsic excitability [117]. On the postsynaptic side, S-nitrosylation can directly affect the activity of NMDARs themselves via a regulatory feedback loop that requires the Grin2a/NR2a-subunit [118], and has additionally been shown to functionally modify a plethora of other excitability-related ion channels and receptors (reviewed in [29]). For example, in hippocampal neurons, surface expression and stabilization of GluA2 is regulated via NMDAR/Nos1-dependent S-nitrosylation of N-ethylmaleimide-sensitive factor (NSF), which contributes to the induction of long-term potentiation (LTP) [119]. Similarly, in the retina, NO-mediated S-nitrosylation is thought to regulate activity-dependent synaptic plasticity, but in this particular system through modulation of Ca_v_ function to achieve lower activation thresholds [120,121]. Therefore, NO specifically increases the sensitivity of photoreceptors and bipolar cells to low-intensity stimuli. Besides the S-nitrosylation pathway, this can also be achieved through increased cGMP production via a NO-sensitive sGC [121,122]. At IHC synapses, analogous scenarios could occur, but this remains to be investigated.

Together, a picture emerges in which the differential distribution and specific abundance of NMDAR/Nos1 signaling components may distinctly affect the performance of individual synapses or even entire SGN subpopulations during ongoing and/or recurrent (spontaneous) activity. Depending on the exact molecular composition of each signaling complex, this could site-specifically influence pre- as well as postsynaptic functional properties. It is therefore conceivable that such a mechanism contributes to the structural and functional heterogeneity of IHC synapses that can be observed at hearing onset [7,94,123]. Yet, the local distribution pattern of NMDAR/Nos1 signaling components as well as a functional relevance of this pathway for IHC neurotransmission and developmental maturation of afferent synapses remains to be analyzed. However, the basic components, including Nos1, sGC and PKG, as well as various NO-regulated receptors and ion channels are present in both developing SGNs and hair cells (Figure 4) and hence await individual in-depth experimental interrogations. It also remains to be clarified if NO volume transmission can be triggered by synchronous activation of the afferent innervation during pre-sensory autonomously-generated spontaneous activity waves, and if/how such a process might influence the establishment of synapse size gradients along the pillar/modiolar IHC axis. Future experiments employing long-term patterned optogenetic IHC stimulation regimes [124,125] and/or iGluSnFr imaging for high-throughput optical detection of evoked glutamate release events [123] in combination with pharmacological manipulation of the NO synthesis pathway might help address these issues.

### 3.2. The Cochlear eCB System

**Figure 5:**
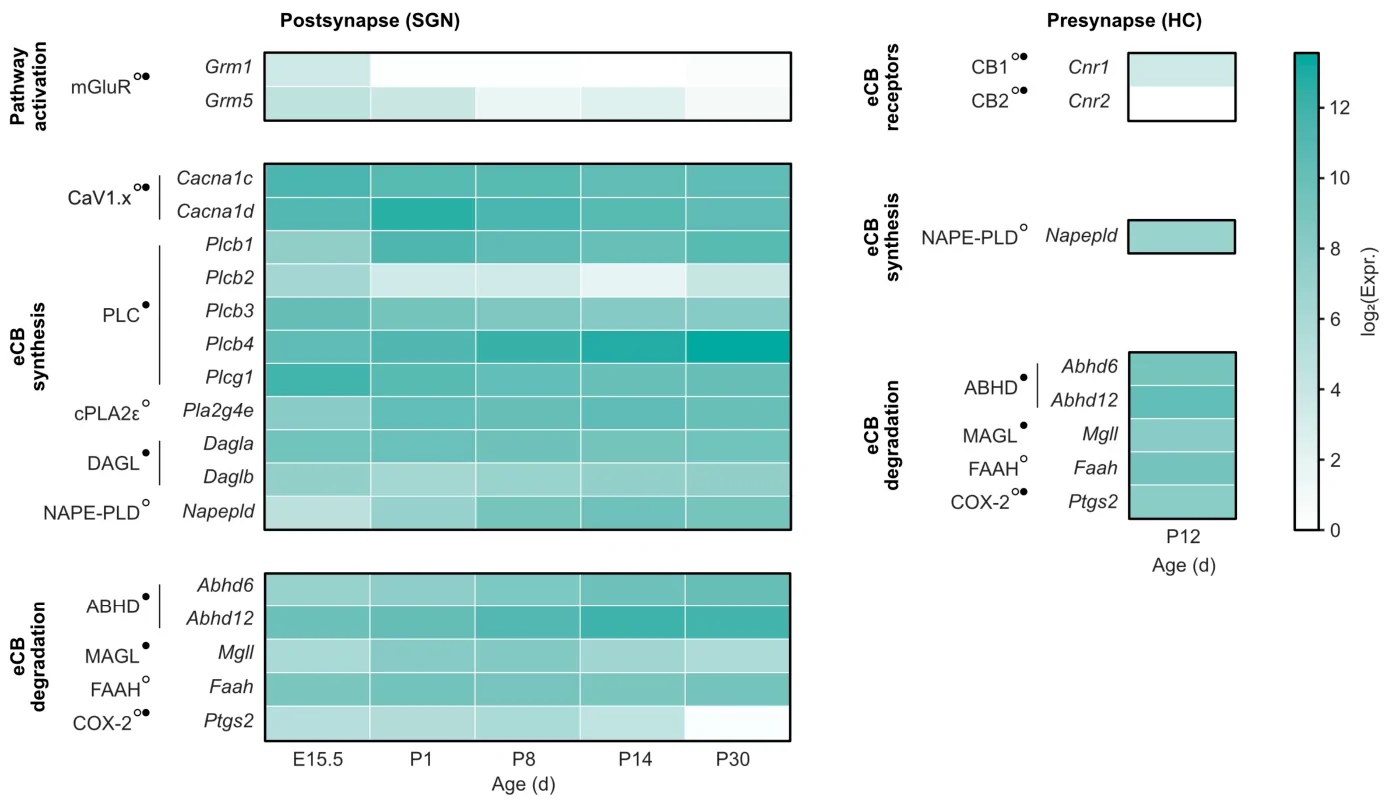
**Differential expression of selected genes associated with retrograde endocannabinoid (eCB) signaling in postsynaptic spiral ganglion neurons (SGNs) and presynaptic hair cells (HCs).** Data highlight cell-type-specific enrichment patterns to estimate a potential contribution of eCB-mediated retrograde communication to the HC–SGN synapse maturation. Genes encode components related to pathway activation, eCB synthesis, degradation and receptors. Color intensity represents transcript abundance. Filled and empty dots indicate involvement in 2-AG and AEA signaling, respectively. Data was acquired using bulk RNA sequencing of manually picked SGNs at different developmental time points (E15.5, P1, P8, P14, P30) and FACS-sorted hair cells at P12 (both cochlear and vestibular) [10].

Postsynaptic class I mGluRs are widely recognized to initiate eCB signaling. For example, a previous study on the calyx of Held synapse of the medial nucleus of the trapezoid body (MNTB) reported mGluR1-dependent inhibition of presynaptic Ca^2+^ influx and neurotransmitter release via retrograde CB1R activation [126]. In the cochlea, a multitude of studies have provided evidence for functional expression of class I mGluRs [127,128,129,130] (Figure 5), which serve to regulate synaptic excitability in various ways: next to a proposed modulatory role in anterograde glutamatergic signaling, mGluR1 has been shown to enhance inhibitory cholinergic feedback from olivo-cochlear efferent terminals, thus further refining pre-sensory spiking behavior and synaptic maturation during early postnatal IHC development [131].

For mGluR-mediated retrograde eCB signaling to play a role in IHC synaptic maturation, the expression of its multi-tiered biosynthesis pathway components, presynaptic receptors and degradation enzymes is a fundamental prerequisite. Indeed, developmental single-cell RNA sequencing profiles indicate abundant expression of a multitude of relevant components—from different PLC isoforms, including PLCβ1 that serves as a coincidence detector for depolarization-induced postsynaptic Ca^2+^ influx and class I mGluR activation [132], to DAGL isoforms and the 2-AG hydrolyzing enzymes of the alpha/beta-hydrolase domain-containing (ABDH) family (Figure 5). Interestingly, expression levels of *Plcb4* (PLCβ4), *Napepld* (NAPE-PLD), *Abhd6* and *Abhd12* (ABHD12) of the same data set showed an increase with age (E15.5 to P30) in SGNs, thus implying potential involvement in maturation-dependent refinement processes. Regarding mGluR-encoding genes, despite their well-established involvement in the initiation of eCB signaling, the data set implies only weak expression, especially for GluR1 (*Grm1*), in SGNs. Similarly, the CB1-encoding *Cnr1* was rather weakly expressed in HCs, and *Cnr2* mRNA levels were (nearly) undetectable (Figure 5). However, immunohistochemical stainings of the eCB receptor complement in rat inner ears detected CB1R and CB2R expression in both hair cell types, inner and outer pillar cells, the stria vascularis and SGN somata [133,134,135]. Moreover, acute pharmacological inhibition of CB2R via trans-tympanic administration of the CB2R inverse agonist/antagonist iodopravadoline (AM630) in mature rats caused a 10–20 dB SPL threshold elevation across the measured frequency range and has hence been proposed to exert a tonic otoprotective function [135]. However, pharmacological activation of CB2R-mediated eCB signaling in the cochlea has so far only been ascribed a protective role in cisplatin-induced toxicity that could potentially be therapeutically exploited to prevent hair cell loss under this treatment regime [134,135].

While still largely anecdotal at this point, the above findings manifest eCB signaling as a potentially powerful retrograde pathway in the cochlear periphery that warrants future investigations, in particular during postnatal maturation but also upon adolescence and adulthood. Future research should aim to (i) assess differential expression of the eCB activation and synthesis components across SGN subtypes, (ii) establish longitudinal nanoscale cannabinoid receptor distribution maps within IHC basolateral membranes, (iii) pharmacologically manipulate IHC exocytosis to dissect the effect of eCB signaling on single-cell synaptic physiology and (iv) test the functional requirement of eCB signaling for adequate synapse maturation. In fact, at this point, the only direct evidence indicating eCB involvement in ribbon synapse assembly stems from a single study on zebrafish inner ear, retina and lateral line neuromast hair cells, which examined *cnr2* loss-of-function mutants. Here, the authors reported an over-representation of immature and dysmorphic synapses at 5 days post-fertilization, a misalignment of pre- and postsynaptic compartments and the misplacement of presynaptic Ca_v_1.3 channels in hair cells of the inner ear and lateral line, which ultimately resulted in impaired evoked acoustic responses [136]. At mammalian ribbon synapses, additional lessons may be learned from the retina, where eCB signaling via CB1R activation is a widespread mechanism for the modulation of neurotransmitter release [137,138,139,140]. Yet again, a role for eCB signaling in retinal ribbon synapse maturation remains to be determined.

### 3.3. Cell Adhesion Molecules at Cochlear Ribbon Synapses

**Figure 6:**
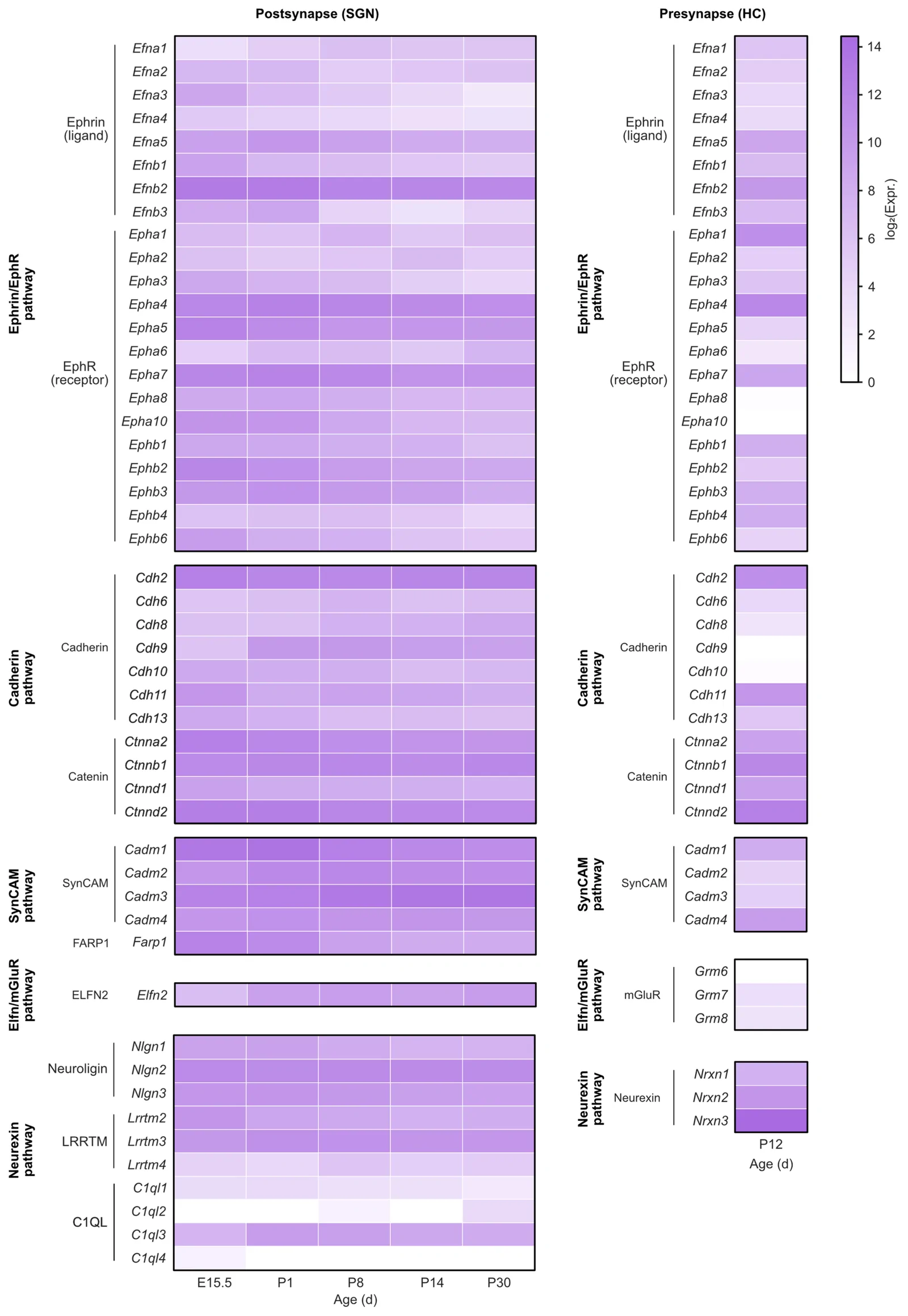
**Differential expression of selected genes associated with retrograde cell adhesion molecule (CAM) signaling in postsynaptic spiral ganglion neurons (SGNs) and presynaptic hair cells (HCs).** Data highlight cell-type-specific enrichment patterns to estimate a potential contribution of CAM-mediated retrograde communication to HC–SGN synapse maturation. Genes encode components related to the ephrin/EphR, cadherin, SynCAM, Elfn/mGluR and neurexin pathways. Color intensity represents transcript abundance. Data was acquired using bulk RNA sequencing of manually picked SGNs at different developmental time points (E15.5, P1, P8, P14, P30) and FACS-sorted hair cells at P12 (both cochlear and vestibular) [10].

In the organ of Corti, various CAMs have been identified that contribute either to sy-napse formation, maturation and/or diversification. Specific roles in pathfinding and terminal branching of SGNs as well as contact formation and maintenance with presynaptic hair cells have been reported for NrCAM [141], semaphorins [142,143,144] and EphR/ephrins [145,146]. Here, the transsynaptic coupling is crucial to coordinate the initial alignment of the presynaptic SV release machinery and postsynaptic receptor and scaffold densities. In addition to the cell adhesion function during synapse consolidation, a subset of CAMs serve a dual role by retrogradely regulating the exocytotic release capacity and protein composition at the AZ of various CNS synapses [70,73]. In this context, an interesting candidate pathway is mediated by presynaptic Nrxns in connection with their diverse set of postsynaptic partners, including Nlgns [147,148], leucine-rich repeat transmembrane proteins (Lrrtm) [77,78], and C1q-like proteins (C1qls) [149]—all of which are expressed in the developing organ of Corti (Figure 6). In fact, Nrxn3 is essential for transsynaptic coupling and synaptic maturation in the mouse cochlea and zebrafish lateral line [150]. Moreover, its postsynaptic binding partners Nlgn1 and Nlgn3 have been identified as regulators of IHC-SGN coupling, with the double knock-out (dKO) showing reduced synapse size and number, increased pre- and postsynapse coupling distance, and increased AMPAR as well as presynaptic ribbon volumes [151]. The dramatic structural defects of the dKO were only partly mirrored by the individual single KOs, indicating functional redundancy or compensation between Nlgn1 and Nlgn3. However, the high and developmentally increasing mRNA levels of Nlgn3, plus an elevated sensitivity to noise similar to the dKOs, highlights Nlgn3 as a possible driver of transsynaptic signaling at the IHC-SGN synapse (Figure 6) [151]. Although this demonstrates a significant role for the Nrxn-Nlgn connection at auditory ribbon synapses, genetic disruption of Nrxn3 as well as Nlgn1/3 dKO “only” leads to a ~25% loss of formed synapses in both conditions [150,151]. This suggests the presence of other synaptic CAMs that can compensate for this loss and additionally raises questions regarding differential SGN subtype distributions of the respective postsynaptic components that will have to be addressed in future experiments. In this context, postsynaptic Lrrtm and C1qls should also be assessed as alternative binding partners for presynaptic Nrxn in IHCs. For example, recent work from the retina identified a key role of Lrrtm4 in the assembly of rod photoreceptor ribbon synapses [152], while Lrrtm3 is highly expressed in SGNs (Figure 6). In contrast, the data regarding a potential contribution of C1ql1 to synapse assembly in the cochlear periphery are still sparse and controversial at this point [153,154]. It will therefore have to be revisited to establish a clear contribution to afferent synapse stabilization and/or retrograde modulation. Furthermore, the N-cadherin pathway also employs presynaptic Nrxn through association of postsynaptic N-cadherin with Nlgn, thereby regulating SV recruitment and replenishment at conventional synapses [155]. In fact, cadherins as well as their intracellular interaction partners catenins are widely expressed in the organ of Corti (Figure 6), thus rendering them, in addition to their conventional function in synapse adhesion, as additional interaction partners for Nrxn in retrograde signaling.

Finally, the current lack of experimental data on other CAMs with known retrograde signaling functions, which nevertheless are developmentally expressed in IHCs and/or SGNs, lead us to speculate on their potential roles in synaptic maturation: for example, the coordination of IHC exocytosis could be mediated though a connection by Nlgn3 or Elfn2 to presynaptic class III mGluRs, for which expression has been reported at auditory ribbon synapses [82,85,156]. Another possible candidate could be SynCAM1, which was shown to contribute to rod ribbon synapse formation in the retina [157] and regulates presynaptic release probability and AZ remodeling at hippocampal synapses through its signaling cascade with Farp1 and PSD95 [158,159]. Lastly, in addition to its determination of the HC innervation pattern, the EphB/ephrinB pathway presents another potential candidate for retrograde signaling in the organ of Corti [146]. Thus far, the only involvement of EphB/ephrinB in retrograde signaling has been proposed through its implication in NMDAR-independent LTP in the hippocampus [160,161]. Whether a comparable potentiation mechanism is present in developing IHCs, either through presynaptic Ca_v_1.3 or mGluRs, is yet to be established. Altogether, the transsynaptic connection through CAMs is a fascinating field opening up in IHC research. The numerous candidate pathways highlight the different avenues to investigate the exact contribution of CAM-dependent retrograde signaling in synapse specification and diversification in the organ of Corti. However, it is also worth highlighting that determining whether a pathway is directly conducting retrograde signaling or rather indirectly employing a different pathway through modulating synaptic activity or connectivity is generally not a straightforward task. In this context, a recent study investigated the involvement of reticulon 4 (RTN4) or so-called ‘Nogo’ receptors in IHC-SGN synapse formation [162]. Here, the loss of RTN4RL2 resulted in enlarged presynaptic ribbons and a lower activation threshold for presynaptic Ca_v_1.3 channels. However, since RTN4s, alike many other CAMs (Figure 6), are synaptic adhesion molecules that are present pre- as well as postsynaptically, it currently remains unclear whether the apparent presynaptic scaling is due to the loss of RTN4-mediated anterograde or retrograde signaling, or secondary effects due to the concurrent loss of AMPAR in the *Rtn4rl*2-KO, which again may trigger some of the aforementioned retrograde signaling pathways.

### 3.4. Effects of Corrupted Retrograde Signaling Pathways on Auditory Performance and Ribbon Synapse Morphology

Currently, our lack of mechanistic understanding regarding retrograde signaling pathways in the cochlea poses a major limiting factor in associating specific retrograde signaling genes with sensorineural hearing loss. In particular, the functional interplay of the distinct pathways, developmental compensation and functional redundancy as well as the expected severity of global loss-of-function phenotypes of such crucial regulatory elements often prevent clear-cut interpretations of the available data. Nevertheless, various studies reported observations that are consistent with transsynaptic regulatory retro-feedback: for example, deletion mutants for the postsynaptic iGluR GluA3 display aberrant presynaptic ribbon morphologies, reduced ribbon volumes as well as the breakdown of the pillar-modiolar ribbon size gradient [163]. Such effects could be transmitted via various retrograde routes, including altered postsynaptic Ca^2+^ signaling and subsequent release of diverse diffusible messengers and/or direct CAM-related signal transmission from the pre- to the postsynapse. Future work will be needed to address this.

Regarding the cochlear eCB system, deletion mutants for CB1R and the 2-AG metabolizing enzyme ABHD12 have been reported and indeed display auditory phenotypes: conditional *Cnr1*-KOs in parvalbumin-expressing GABAergic interneurons show ABR threshold elevations alongside increased latencies and reduced amplitudes of Jewett Wave I-IV for 90 dB SPL click stimulations and high-frequency pure-tone bursts [164]. The authors of the study attributed these phenotypic aberrances to attenuated CNS interneuron function in the central auditory nuclei; however, since SGNs also express parvalbumin, these findings are also consistent with a direct functional impact on SGN physiology that remains to be investigated. In addition, loss-of-function mutations in the lyso-phosphatidylserine lipase ABHD12 have been shown to cause PHARC (“polyneuropathy, hearing loss, ataxia, retinitis pigmentosa, and cataract”) syndrome in humans that is characterized by early-onset, progressive sensorineural hearing loss [165]. As mentioned above, ABHD12 is, alongside MAGL and ABHD6, a key enzyme in the metabolism of the eCB 2-AG. In *Abhd12*-KO mice, while the acoustic startle response was strongly attenuated, basic audiometry failed to detect significant effects on the early Jewett Waves I-III [166]. Future studies will hence be required to further scrutinize hearing capacity in these mice, assess their vulnerability to noise trauma and evaluate functional redundancy via MAGL and/or ABHD6 compensatory upregulation.

In regard to NO signaling, the picture is even less clear. This is partly due to its volatility and the diversity of potential effector pathways that not only fine-tune synaptic function but also regulate vasodilation of nearby blood vessels. In fact, cochlear NO production has been proposed to either protect against [167] or exacerbate excitotoxicity due to noise trauma [103,168]. Yet, Nos1 loss-of-function mutations in mice and humans are associated with mild hearing deficits [169], albeit the exact peripheral contribution to the observed phenotype still remains to be determined. However, in further support of a regulatory role of NO in cochlear function, a recent study identified carboxypeptidase D (CPD) as a potential new deafness gene that causes severe to profound sensorineural hearing loss in humans [170]. CPD is a metallocarboxypeptidase involved in L-arginine processing, which ultimately serves as the substrate for NO production. In this study, biallelic missense mutations leading to CPD deficiency resulted in increased apoptosis during the early developmental phase of the sensory epithelium. While the exact cellular mechanisms remain unclear at this point, in vitro L-arginine supplementation offered a putative therapeutic avenue for the development of future treatments for this condition. However, this still needs to be thoroughly evaluated prior to clinical adoption [170].

Finally, genetic ablation of several CAMs have been shown to negatively impact on hearing function: next to the above-mentioned Nlgn/Nrxn [150,151] and RTN4RL2 [162] phenotypes, which show clear deficits in transsynaptic signaling and pathway assembly, novel candidate molecules include EphA10, which has recently been proposed as a new deafness gene [171]. However, a major caveat with Eph/ephrins in the context of retrograde signal transmission is their documented role in neuronal pathfinding and initial contact formation [145,172]. Hence, the interpretation of knockout phenotypes requires careful analyses of the overall innervation pattern, which in the case of EphA10 loss-of-function is currently still lacking.

In summary, while the exact molecular/cellular mechanisms for many of the above-mentioned phenotypes still remain to be experimentally determined, they nevertheless illustrate the importance of retrograde signaling pathways in cochlear development and adult function and should serve as a blueprint for future analyses.

## 4. Conclusions and Future Perspectives

Retrograde transsynaptic signaling mechanisms in the cochlea remain largely under-explored and thus present a major knowledge gap in our current understanding of cochlear ribbon synapse maturation. This shortcoming needs to be addressed, and future studies should seek to establish SGN subtype-specific expression profiles of the different pathway components across the developmental timeline. Moreover, special attention should also be dedicated to identifying tonotopic differences in the individual pathway expression patterns. This might help explain frequency-specific functional differences between low- vs. high-frequency mechanisms of synaptic sound encoding. Of note, since mRNA levels are often not proportionally translated into protein levels, the scRNAseq expression data will have to be confirmed by immunolocalization and functional characterization and extended by the identification of relevant effector molecules. Finally, it will be equally important to dissect the functional interplay between the individual pathways—as shown, for example, in case of eCBs and NO in the brain [173] or BDNF/TrkB and the eCB system at calyceal synapses of the brainstem [174]. We expect that novel methods to hijack and manipulate the intrinsic excitability program will help address these questions in the foreseeable future [124,125].

## Data Availability

This short review contains replotted original RNA sequencing data from ref. [10] that is publicly available (GEO ID GSE132925) and was accessed through gEAR [175].
